# Radiation-induced oral mucositis as an immune hub: from innate immune sensing to adaptive immune dysregulation

**DOI:** 10.3389/fimmu.2026.1905406

**Published:** 2026-10-15

**Authors:** Xiaoguang Xue, Shenghong Qiu, Jianxiao Zheng, Yilin Kong, Lei Gao, Wenting Zhan, Weiwei Guo, Jixin Chen, Longmei Li

**Affiliations:** 1 Department of Radiotherapy, The Second Affiliated Hospital of Guangzhou University of Chinese Medicine, Guangzhou, Guangdong, China; 2 State Key Laboratory of Traditional Chinese Medicine Syndrome, The Second Affiliated Hospital of Guangzhou University of Chinese Medicine, Guangzhou, China

**Keywords:** DAMPs, immune cell dysfunction, immune homeostasis, microbiome dysbiosis, radiation-induced oral mucositis

## Abstract

Radiation-induced oral mucositis (RIOM) has traditionally been viewed as a cytotoxic injury to rapidly proliferating epithelial cells caused by radiotherapy. Recent studies have revealed that RIOM is fundamentally a pivotal radiotherapy-triggered, damage signal-driven dysregulation event of local and systemic immune homeostasis. The severity of RIOM dynamically and directly affects patients’ oral health-related quality of life (OHRQoL), serving as an intuitive reflection of their local immune-inflammatory status. Key immunological events include the release of unique damage-associated molecular patterns (DAMPs) by radiotherapy, which actively initiate and shape the intrinsic immune recognition program; functional shifts and imbalances in critical immune subsets such as neutrophils, macrophages, and T cells, forming the core circuitry of sustained injury and impaired repair; and the chronic immune microenvironment, which not only drives local pathology profoundly influencing systemic antitumor immune responses and long-term complications. This review aims to establish the emerging interdisciplinary framework of radioimmunomucosology, providing a novel perspective for optimizing comprehensive cancer therapy. Additionally, systematic studies have revealed that radiotherapy induces predictable ecological dysbiosis in the oral microbiome, characterized by depletion of commensal bacteria and expansion of opportunistic pathogens. This suggests that RIOM involves radiation-triggered disruption or confrontation between host immunity and commensal microorganisms.

## 1 Introduction

Radiation-induced oral mucositis (RIOM), as the most prevalent dose-limiting toxicity in head and neck radiotherapy, has long been considered merely a passive physical barrier disruption caused by the depletion of epithelial stem cells (1-2). This limited understanding has severely hindered the innovation of prevention and treatment strategies (3-4). Radiotherapy is not only a DNA-damaging agent but also an immunomodulatory process that includes the release of tumor neoantigens, the activation of innate immune sensing, and the promotion of antitumor immunity (5-6). However, during radiation, the oral mucosa is subjected to equally or even more intense immunomodulatory effects, yet RIOM has been overlooked (7-8). According to emerging theories, RIOM is a disruption of homeostasis in the unique immune niche of the oral cavity and can be used as an ideal model for studying localized intense immune perturbations (9-10). With the increasing use of immune checkpoint inhibitors (ICIs) in combination with chemoradiotherapy, evaluating their impact on RIOM has become critically important (11, 12). Zou et al. (2025) reported that although anti-PD-1 therapy did not increase the overall risk of severe RIOM, it significantly elevated systemic inflammatory states in younger patients (<45 years), those receiving TPF/X or TP induction chemotherapy, and patients with a preradiotherapy NLR ≥2.5 (11). This discovery indicates that RIOM is not an isolated local toxicity event but rather a response of the systemic immune system to radiation damage (13, 14). Therefore, it is essential to re-examine the role of RIOM in combination therapies and regard it as a critical node for predicting and managing overall immune-related toxicity (11, 13, 15).

Under modern precision radiotherapy, RIOM remains unavoidable. Its occurrence follows a clear dynamic progression pattern, which starting at 19 Gy, peaking at 30 Gy, with a threshold at 37.7 Gy, and its severity is directly and closely related to the patient’s nutritional status (16–18). A clinical study showed that although all patients developed mucositis by the end of treatment, no grade IV mucositis occurred, and there were no treatment interruptions due to mucositis (19). Notably, advancements in radiotherapy technology, while precisely targeting tumors, have enabled researchers to reveal the dose–response relationship and clinical trajectory of RIOM (14, 17, 18). Among 55 patients with locally advanced oral squamous cell carcinoma who underwent radical VMAT, although all developed oral mucositis, the incidence of severe (≥grade 3) cases was 50.9%, and other acute toxicities were relatively manageable (17). The median total treatment time in this study was 43 days, and high treatment compliance may be related to early tumor control rates (17). RIOM is the most significant acute toxicity, and its occurrence patterns and severity can be meticulously documented under standardized treatment, providing a high-quality clinical benchmark for establishing dose–response models and evaluating the effectiveness of supportive therapies (14, 17, 18). The retrospective study by Gobbi et al. (2025) offers comprehensive data supporting this relationship (17). In head and neck cancer patients who underwent VMAT, the incidence of severe (grades 3–4) RIOM was as high as 61.8%, with a median cumulative dose threshold of 37.7 Gy, and it was significantly associated with concurrent chemoradiotherapy (17). From a clinical epidemiological perspective, this study confirms that RIOM is not a static event but rather a dynamic process that changes synchronously with dose accumulation and directly and profoundly affects patients’ nutritional status and quality of life (17, 20, 21). These findings indicate that RIOM is a radiation-induced immune-inflammatory syndrome with distinct kinetic characteristics, and its severity is a direct clinical indicator of local immunopathological activity, making active management and prevention crucial (14, 16, 17). The assessment tools for RIOM are also evolving, integrating clinician-reported outcomes with patient-reported outcomes (22-23). The RTOG/EORTC scoring system and OMI provide standardized tools for clinical research and practice (24–26).

RIOM has long been misinterpreted as a simple epithelial injury, but recent theories indicate that RIOM essentially represents a “targeted destruction” of the oral immune niche by radiation, and it is a localized immune-inflammatory syndrome with distinct kinetic characteristics (10). With the combined application of immunotherapy and radiotherapy, RIOM has been confirmed to reflect systemic immune status (11-13). Specific populations exhibit significantly altered risks after combined immunotherapy, indicating that RIOM no longer exists in isolation (7, 11). Under modern precision radiotherapy, while RIOM remains unavoidable, its occurrence follows dynamic patterns that are synchronized with the cumulative radiation dose, and its severity directly correlates with patients’ nutritional status and quality of life (17, 18, 20). Collectively, this evidence suggests that RIOM is a radiation-triggered, dynamically evolving immunopathological process that must be actively monitored and managed within the holistic framework of combination therapy (7, 12, 14).

## 2 Immunopathology of RIOM: initiation, progression, and ripple effects

Radiation-induced oral mucositis involves not only simple epithelial injury but also a series of pathological cascade reactions initiated by radiation, dominated by the immune system, and continuously amplified (12, 27). This chapter systematically elaborates on the immunopathological progression of RIOM: radiation acts as an “immune initiator,” triggering inflammatory signals by inducing cell death and the release of DAMPs (8, 28, 29); hyperactivation and dysfunction of innate and adaptive immune cells drive tissues from protective repair to destructive damage (5, 30, 31); and local immune events, through systemic ripple effects, impact antitumor efficacy and patients’ long-term quality of life, collectively forming the biological basis of RIOM (11, 13, 32). This pathological process involves a complex cytokine network and multicellular interactions (30, 33, 34).

### 2.1 Radiation injury: the initiation and “immune priming” of RIOM

The energy deposition of radiotherapy first triggers a “catastrophe” at the molecular and cellular levels, and these endogenous danger signals constitute the starting point of the immune response (8, 9, 35). Radiation directly causes nuclear DNA and mitochondrial DNA breaks, damages cell membranes and organelles, and induces the release of a series of DAMPs whose release varies in space and time (28, 36, 37). High mobility group box 1 (HMGB1) escapes from the nuclei of damaged cells and binds to TLR4 and the receptor for advanced glycation end products, leading to strong proinflammatory signals (28, 29, 38). Adenosine triphosphate (ATP) leaks extensively from ruptured cells, acting as a chemoattractant that recruits neutrophils and macrophages through purinergic receptors and may activate the NLRP3 inflammasome (35, 39). In contrast to chemotherapy or surgical trauma, radiation-induced DNA double-strand breaks generate unique nucleoplasmic fragments and chromatin modifications, which may be recognized by cytoplasmic DNA sensors, thereby activating the stimulator of interferon genes (STING) pathway (6, 40). DAMPs are crucial in antiviral and antitumor immunity; however, in RIOM, their aberrant signaling within the mucosa may lead to persistent type I interferon responses, exacerbating tissue damage (6, 39, 41).

Radiation-induced cell death does not occur through a single mode, and its immunogenicity determines the direction of subsequent reactions (29, 42, 43). Immunogenic cell death (ICD) is characterized by exposure to calreticulin and the release of ATP and HMGB1, which efficiently activate dendritic cells. However, in RIOM, excessive ICD may lead to uncontrolled inflammatory storms (38, 42, 44). Pyroptosis, a highly inflammatory form of programmed cell death mediated by gasdermin proteins, may be activated in epithelial cells after radiation, resulting in the intense release of IL-1β and IL-18 (38, 39). In contrast, apoptosis is generally considered immunologically silent, but if efferocytosis is impaired, secondary necrosis can occur during clearance failure, leading to the release of DAMPs (38, 43). Under persistent radiation damage, clearance cells such as macrophages may become overloaded or dysfunctional, leading to the accumulation of large amounts of dead cell debris. Untimely cleared cell debris acts both as an antigen that continuously stimulates the immune system to attack itself and as a source of persistent inflammatory signals (35, 38). Under the sustained influence of these dual factors, acute inflammation evolves into chronic inflammation (7, 12, 38).

### 2.2 Immune dyshomeostasis: the core driver from local inflammation to tissue destruction

The damage signals that are released recruit and activate innate and adaptive immune cells, but their responses often shift from protective repair to destructive effects on host tissues (5, 7, 30). As the first immune cells to reach the injury site, neutrophils act to control the injury site and potential infections by releasing reactive oxygen species and proteases and by forming neutrophil extracellular traps (NETs) (5, 45, 46). However, in RIOM, NETosis may become excessive and uncontrolled. Excessive NETs not only directly damage epithelial and endothelial cells but can also function as DAMPs, further amplifying inflammation and forming a positive feedback loop (5, 45, 46). Persistent activation leads to functional exhaustion of neutrophils, but their physical presence and residual inflammatory secretions still contribute to microenvironment disruption (5, 45, 46). The dynamic polarization of macrophages is a critical switch in the progression of RIOM (27, 47). Early on, classically activated M1 macrophages dominate, producing TNF-α, IL-6, IL-1β, etc., to clear necrotic tissue and pathogens, but excessive M1 responses exacerbate tissue edema and destruction (27, 47). The repair phase is theoretically dominated by alternatively activated M2 macrophages, which promote angiogenesis and matrix remodeling (27, 47, 48). However, under continuous radiation stimulation, this M1-to-M2 transition may occur prematurely, excessively, or become dysregulated. Abnormal M2 polarization not only fails to effectively terminate inflammation but also induces fibroblast activation through the secretion of large amounts of cytokines such as TGF-β and PDGF, which contributes to submucosal fibrosis (31, 47, 48). Additionally, there are fundamental differences in function and regenerative contributions between tissue-resident macrophages and circulating monocyte-derived macrophages, with the former likely being more critical for maintaining local homeostasis (27, 47, 49). Animal model studies provide an essential foundation for understanding immune cell dynamics (50-51).

The involvement of adaptive immunity reflects the chronicity and complexity of RIOM (31, 46). Disruption of the Th17/Treg balance is among the core features (31, 52). Locally released IL-6, TGF-β, and fungal antigens (such as Candida) strongly promote Th17 differentiation, resulting in the production of IL-17 and IL-22 (31, 52, 53). IL-17 further induces neutrophil recruitment, resulting in a vicious cycle; IL-22 promotes epithelial repair at low concentrations but may inhibit differentiation and promote hyperplasia under chronic high concentrations (31, 52). Moreover, the number of Treg cells may be reduced or Treg cells may be functionally suppressed; thus, the excessive responses of Th17 and effector T cells cannot be restrained (31, 52). Cytotoxic CD8^+^ T cells are recruited to eliminate virus-infected or severely damaged epithelial cells, but their cytotoxic effects undoubtedly exacerbate epithelial denudation (31, 46). More importantly, these immune responses generate long-lived tissue-resident memory T cells, leaving the mucosal tissue in a “hypersensitive” state that overreacts to subsequent minor stimuli (31, 46, 52). The refractoriness of RIOM ultimately stems from the self-reinforcing vicious cycle formed between immunity, the epithelium, and the microbiome, and the dysregulation of the aforementioned immune microenvironment is the core driving force of this “malignant triangle” (7, 54). Dysregulation of protective signaling pathways, such as keratinocyte growth factor, also contributes to this process (49, 55, 56). The core immune and microbial pathological mechanisms of RIOM are summarized in **Figure 1**.

**Figure 1:**
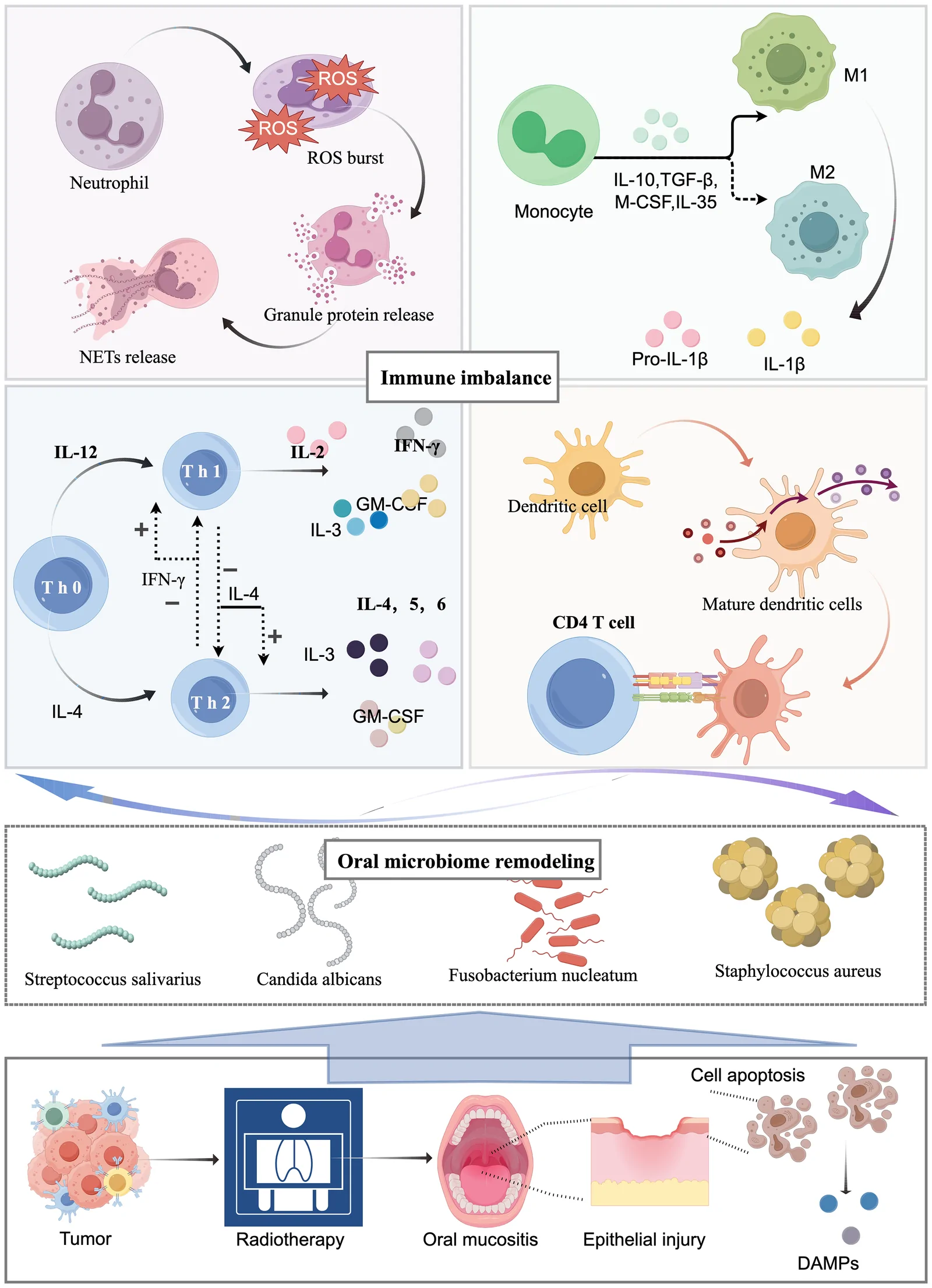
Schematic diagram of immune cell dysfunction and pathological mechanisms in radiation-induced oral mucositis.

### 2.3 Ripple effects of local immune events on systemic treatment paradigms

RIOM should not be regarded as an isolated local event, because its immune-related consequences may affect systemic treatment outcomes (11, 32). The occurrence of severe RIOM in patients may reflect their inherent or radiotherapy-induced propensity for excessive immune responses (11, 14). Interestingly, this intense local immune activation may share upstream mechanisms with the potential antitumor “abscopal effect” after radiotherapy (6, 41). Alternatively, the excessive, ineffective inflammatory response might deplete immune resources and thereby hinder systemic antitumor immunity (15, 57). The severity of RIOM might be a biomarker for predicting the overall type of immune response a patient will have to radiotherapy or combined immunotherapy (11, 14). Clinical observations suggest that dynamic changes in OHRQoL scorescan sensitively capture the severity of this immunopathological process (22). This speculation raises an urgent clinical question: does severe RIOM alter the tumor microenvironment or affect systemic immune status, thereby reducing the efficacy of subsequent immune checkpoint inhibitor (ICI) therapy (11, 15)? Animal models and retrospective clinical analyses are beginning to explore this question, with one hypothesis suggesting that RIOM-related systemic inflammation and immune cell exhaustion may impair the immune system’s ability to mount effective responses to neoantigens (11, 57). The economic burden and treatment interruption risks associated with RIOM cannot be overlooked (20, 58).

In addition to the effects of current treatments, the immune memory induced by RIOM may lead to long-term changes (32, 59). Persistent local inflammation and recurrent infections may accelerate the aging of the immune system, manifested as reduced diversity of the T-cell repertoire and accumulation of senescent T cells (59). Furthermore, immune responses targeting exposed self-antigens could increase the risk of autoimmune diseases in the long term (15). These long-term immunological changes result in prolonged physical functional limitations and reduced quality of life for patients (21, 22, 32). Chronic pain and functional limitations are common long-term burdens following RIOM and severely affect survivors’ quality of life (19, 21, 22). Late complications such as dry mouth, dysphagia, and fibrosis further decrease patient survival (60, 61).

## 3 Radiotherapy-driven microbial–immune interaction network

Radiation-induced oral microbiome dysbiosis is not merely a complication but also a core driver that actively promotes the chronicity and severity of mucositis (54, 62, 63). This chapter systematically elaborates on the radiation-driven microbial,immune interaction network. First, metagenomic studies have revealed the specific remodeling landscape of the oral microbial community after radiotherapy and the identification of “key pathogenic bacteria” such as Fusobacterium nucleatum as accelerators of the pathological process (62, 64, 65). In-depth analysis elucidates how the dysregulated microbiota, through mechanisms such as the Candida-IL-17 axis and removal of immune “brakes,” forms a self-reinforcing vicious cycle with the host immune system (52, 54, 66). Breakthrough progress from the latest phase III clinical trials provides robust clinical evidence for microbiome-targeted precision intervention strategies, marking the transition of this field from mechanistic exploration to clinical translation (67-68). Dynamic changes in the oral microbiome can be precisely tracked through longitudinal sampling (69, 70).

### 3.1 Radiation-driven remodeling of the oral microbiome

Metagenomic studies have shown the intricate characteristics of oral microbiome dysbiosis following radiotherapy (54, 62, 66). A comprehensive review of 62 studies reported that radiotherapy induces characteristic alterations in alpha diversity: species richness may increase, but evenness significantly declines, indicating that microbial communities become dominated by a few species (62). Specific trends manifest at three levels: first, the loss of commensal bacteria, with foundational taxa such as Streptococcus, Prevotella, and Rothia, which are critical for maintaining pH homeostasis, competing with and inhibiting pathogens, and regulating local immune tolerance, showing significant decreases (62, 63); second, the rapid proliferation of opportunistic pathogens, with Candida and Staphylococcus aureus emerging as the most prevalent opportunistically expanding genera (62, 71), and their overgrowth not only reflects secondary infections but also involves cell wall components acting as potent innate immune agonists that persistently activate pathways such as TLR2/4 and amplify Th17-type inflammatory responses (52, 62, 71); and finally, the colonization of “foreign” pathogens, where genera typically residing in the gastrointestinal tract or skin, such as Enterococcus, Klebsiella, and Pseudomonas, are frequently detected in the oral cavity after radiotherapy (15, 62, 72). This breach of the “microbial geographical barrier” indicates systemic immune surveillance failure and sets the stage for life-threatening bacteremia (72). The oral microbiota is closely linked to systemic health (57, 59).

Recent longitudinal studies have provided strong evidence for the “critical pathogen hypothesis,” shifting the focus of microbial dysbiosis from general community characteristics to specific pathogens with well-defined pathogenic mechanisms (64, 73, 74). An increase in the relative abundance of Fusobacterium nucleatum after radiotherapy was significantly positively correlated with the severity of mucositis (64, 73, 74). Notably, the strongest association was not with absolute abundance but with post-radiotherapy growth rate, illustrating the concept of a “critical pathogen” (64, 73). Even if the initial abundance is low, abnormal proliferation under disturbance is sufficient to drive pathological progression (64, 65). Mechanistic studies showed that Fusobacterium nucleatum activates the TLR4-NF-κB pathway in epithelial cells through lipopolysaccharide (LPS) in its outer membrane vesicles, leading to the massive release of proinflammatory cytokines such as IL-6, IL-8, and TNF-α. This recruits and hyperactivates immune cells such as neutrophils, exacerbating tissue damage (64, 65). Additionally, Fusobacterium nucleatum can synergize with genera such as Capnocytophaga and Parvimonas to form biofilms, increasing antibiotic resistance and pathogenicity and thereby amplifying its pathological effects (64, 65). Radiotherapy itself may increase the virulence of certain fungi (75).

### 3.2 Microbial-driven mechanisms of immune deterioration

Changes in the microbiome are not passive consequences but actively contribute to the chronicity and severity of RIOM (52, 54, 62). Taking the Candida-IL-17 axis as an example, the expansion of Candida strongly promotes Th17 cell differentiation and activation. The secreted IL-17 further induces neutrophil recruitment, forming a self-reinforcing loop that proceeds from fungal colonization to Th17 activation, then to neutrophil inflammation, followed by mucosal damage, and finally to increased susceptibility to colonization (52, 62, 71). Clinical cases frequently show Candida infection accompanied by severe mucositis and pain (22, 71). Simultaneously, the loss of commensal Streptococcus results in the loss of a critical immune regulatory mechanism. Under normal conditions, Streptococcus inhibits Candida growth through the secretion of hydrogen peroxide, bacteriocins, etc., and helps maintain immune balance by regulating regulatory T-cell function (62). Its loss not only removes suppression of pathogens but also weakens the restraint on excessive inflammation (62). This dysregulated microbiota continuously stimulates the immune system via pattern recognition receptors, exacerbating inflammatory responses and ultimately driving the chronic progression of RIOM (54, 62, 65). Crosstalk between the oral microbiome and the gut microbiome may amplify systemic effects (53, 70).

The “critical pathogen hypothesis” and microbiome-targeted intervention strategies were supported by a landmark phase III clinical trial in 2025 (76). A rigorously designed randomized controlled trial by Liao et al. (2025) demonstrated that using mupirocin nasal ointment for Staphylococcus aureus decolonization significantly reduced the incidence of severe RIOM in nasopharyngeal carcinoma patients who underwent chemoradiotherapy from 47.7% to 22.7% (76). This breakthrough is reflected in three aspects: local nasal intervention can produce a “cross-compartment effect,” significantly reducing oral S. aureus colonization rates, highlighting the importance of an interconnected upper respiratory microbiota in RIOM pathology (76); the use of precise “narrow-spectrum” antimicrobial agents effectively avoids the ecological disasters potentially caused by broad-spectrum antibiotics, providing a model for “precision ecological remodeling” (76); and the intervention yields clear clinical benefits, including significantly alleviating patient pain and dysphagia (76). This study not only established Staphylococcus aureus as one of the key pathogenic drivers of RIOM but also moved microbiome-targeted therapy from theory to practical clinical application (67-68). The value of prophylactic antifungal therapy has also received partial research support (77). The key immune cell abnormalities, pathological effects, and intervention targets in RIOM are summarized in **Table 1**.

**Table 1:** Major immune effector cells, functional characteristics, and potential intervention targets in RIOM.

Cells/subsets	Key changes	Pathological effects	Potential intervention directions	References
Neutrophils	Early massive infiltration, increased NETs formation	ROS, proteases, and histones trigger secondary tissue damage	Inhibit excessive NETosis; optimize local anti-infection	(5, 45, 46)
Macrophages	M1 dominance persists, insufficient M2 conversion	Sustain TNF-α/IL-1β axis and inhibit repair	Promote polarization rebalance; block inflammasome	(7, 27, 47, 48)
Th17 Cells	IL-17/IL-22 was upregulated	Enhance neutrophil recruitment and maintain chronic inflammation	Regulate Th17/Treg balance	(12, 31, 52)
Treg Cells	Reduced quantity or function	Weakened “braking” capacity against excessive inflammation	Restore immune homeostasis	(12, 31, 52)
CD8^+^ T Cells	Enhanced stress-induced killing	Clear damaged cells while exacerbating epithelial denudation	Reduce bystander injury	(31, 46)

RIOM, radiation-induced oral mucositis; NETs, neutrophil extracellular traps; ROS, reactive oxygen species; Tregs, regulatory T cells.

## 4 Treatment strategies for RIOM: multidimensional interventions and precision management

Building on an in-depth understanding of the immunopathological mechanisms of RIOM and microbial interaction networks, intervention strategies are shifting from single symptom management to multidimensional precision approaches (67). This chapter systematically outlines the RIOM treatment strategy framework: at the immunomodulatory level, focusing on targeted interventions from upstream oxidative stress to downstream effector cytokines (6, 67, 78); at the microbiome–immune axis level, exploring regulatory strategies to reshape microbial ecology through “addition” and “subtraction” and to disrupt vicious cycles (62, 76, 79); and at the clinical management level, emphasizing biomarker-based risk stratification and preemptive personalized prevention (11, 14, 21). The integration of these multidimensional strategies is moving RIOM management toward a new era of mechanism-driven, precision-oriented clinical translation (14, 67, 76). International clinical practice guidelines provide critical foundations for standardized management (80-81).

### 4.1 Targeted intervention strategies based on immune pathways

Targeting innate immune sensors: In the early stages of the cytokine storm, the use of NLRP3 inflammasome inhibitors or STING pathway modulators may block DAMP-driven hyperactivation (6, 38, 39). Regarding the oxidative stress events that occur earliest in radiation injury, a superoxide dismutase mimetic, Avasopasem manganese, has achieved success in phase III clinical trials (67, 82). The ROMAN trial reported by Anderson et al. (2025) demonstrated that intravenous infusion of Avasopasem prior to each radiotherapy session significantly reduced the incidence of severe RIOM (54% vs. 64%) and the median duration (8 days vs. 18 days) in head and neck cancer patients who received concurrent chemoradiotherapy, and it also delayed its onset (67, 82). This drug mitigates initial oxidative damage and subsequent inflammatory signaling cascades by rapidly converting radiation-generated superoxide into hydrogen peroxide at the source (67, 82). It is the first drug proven effective in phase III trials to exert mucosal protective effects by clearing key DAMP precursor substances, marking a major breakthrough in the “upstream intervention” strategy (67, 82). Regulatory cell therapy: Exploring localized reinfusion of *in vitro*-expanded Treg cells or M2-type macrophages to restore immune balance (31, 46, 48). Cytokine-targeted therapies: Monoclonal antibodies or inhibitors targeting key proinflammatory cytokines such as IL-1, IL-6, and IL-17 may precisely alleviate pathological inflammation (30, 31). Anti-inflammatory mouthwashes such as benzydamine hydrochloride have shown certain efficacy in clinical applications (83-84).

### 4.2 Regulation strategies based on the microecology–immune axis

Based on a profound understanding of the dysbiosis patterns of the microbiome after radiotherapy, the RIOM intervention strategy is shifting from broad-spectrum antimicrobial agents to precise ecological remodeling (62, 76, 79). Furthermore,targeted probiotics exert protective effects by restoring competitive exclusion and immune regulatory functions; studies have shown a negative correlation between Lactobacillus abundance and the severity of mucositis (64, 79, 85). Furthermore, multiple RCTs have confirmed that natural products can effectively reduce the severity and pain scores of RIOM (68, 78, 86); topical agents such as manuka honey, aloe vera, and Scutellaria baicalensis extract work through anti-inflammatory and microecological maintenance mechanisms (78, 87, 88), and systemic agents such as oral curcumin capsules alleviate symptoms such as dysphagia via systemic anti-inflammatory and antioxidant mechanisms (78, 86, 89). In terms of “subtraction,” antifungal strategies need to balance immune modulation (22, 71, 77). A phase III study by Liao et al. (2025) provided landmark evidence for precision antimicrobial therapy: intermittent use of mupirocin nasal ointment to selectively eradicate Staphylococcus aureus reduced the incidence of severe RIOM from 47.7% to 22.7% (relative risk 0.48), and through the “trans-cavity effect,” it simultaneously decreased oral colonization rates, perfectly aligning with the “selective antimicrobial” concept,efficiently eliminating key pathogens while minimally impacting the overall microbiota (76). This stands in stark contrast to broad-spectrum antibiotics that disrupt the ecosystem, offering a model for developing narrow-spectrum antimicrobial therapies targeting specific pathogens such as Fusobacterium nucleatum (64, 76). Photobiomodulation therapy, as a physical intervention, has accumulated substantial evidence for preventing and treating RIOM (90-91), and handheld devices also demonstrate promising applications (91-92).

Jia et al. (2025) further revealed the underlying mechanism of the “addition” strategy: a plant complex bidirectionally modulates the oral microbiome,increasing the abundance of beneficial bacteria such as Lachnospiraceae while reducing the abundance of pathogenic bacteria such as Fusobacterium nucleatum,thereby inhibiting overactivation of the oral mucosal TLR4/NF-κB pathway, decreasing proinflammatory M1 macrophage polarization, and ultimately alleviating RIOM (68, 78, 79). This study organically integrates microbiome remodeling, innate immune regulation, and macrophage function modulation, forming a complete microbiota-immune intervention chain, providing critical evidence for the cutting-edge strategy of restoring immune homeostasis by regulating overall microbial ecology (68, 78, 79). Additionally, maintaining oral barrier integrity to prevent abnormal convergence of oral and gut microbiota (53, 62) is equally important for preventing systemic infections and distant immune dysregulation (49, 72). Nutritional supplements such as hyaluronic acid and glutamine have also been shown to have mucosal protective effects (93-94). Novel delivery systems such as oral patches containing Streptococcus lactis are under development (79, 95).

### 4.3 Biomarker-based Risk Stratification and Precision Management

Utilizing tools such as single-cell sequencing and microbial metagenomics to identify high-risk patients before treatment and longitudinally correlating patient-reported clinical outcomes with immune-microbiome data holds promise for determining whether specific dysregulation features can predict severe pain and a decline in quality of life, thereby driving a paradigm shift from “reactive treatment” to “precision prevention” (14, 22, 64). Zou et al. (2025) identified high-risk factors for RIOM under anti-PD-1 therapy and developed and validated a nomogram prediction tool with a C-index >0.72, marking the advent of the risk stratification era (11). Integrating Liao et al.’s (2025) precision decolonization intervention (76) and Anderson et al.’s (2025) upstream immune protection strategies (67), future clinical pathways could evolve into a pretreatment model screening of high-risk patients, early initiation of targeted prevention, and achieving a transition from “equal treatment” to “preemptive” precision medicine (11, 14, 67). The implementation of structured oral care protocols can significantly improve patient outcomes (96–98).

We propose redefining RIOM as an active immunopathological process initiated by the release of DAMPs transmitted through innate immunity, leading to immune cell dysfunction and interactions with damaged epithelium and key pathogenic bacteria (8, 54). On this basis, we suggest establishing the interdisciplinary field of radioimmunomucosology, with core directions including mapping the immune–microbial dynamic atlas, developing spatiotemporally specific immune–microbial modulators, and prospectively integrating immune and microbial management into cancer treatment plans (62, 67). The three high-level studies by Zou (11), Anderson (67), and Liao (downstream blockade) (76) in 2025 outline future clinical blueprints from different dimensions pretreatment risk stratification, combination with targeted oxidative stress protectants, and precise decolonization based on microbial screening. This multimodal personalized prevention strategy is expected to optimize antitumor efficacy while ensuring quality of life; thus, multidisciplinary collaboration is urgently needed (11, 14, 67). Pain management is a critical aspect of RIOM symptomatic treatment, with local anesthetics and opioid mouthwashes providing effective relief (99–101). The mechanism positioning and clinical objectives of common and emerging intervention strategies for RIOM are summarized in **Table 2**.

**Table 2:** Mechanism positioning and clinical objectives of common and emerging intervention strategies for RIOM.

Strategy type	Representative measures	Main targets	Main clinical objectives	References
Basic Supportive Care	Oral care, analgesia, nutritional support	Symptom control and secondary injury prevention	Alleviate pain, maintain oral intake	(20–22)
Upstream oxidative stress control	Photobiomodulation, SOD mimetics	ROS and DAMPs precursors	Delay occurrence, shorten duration	(67, 82)
Immune regulation	Inflammasome/cytokine-related modulation	NETs, NLRP3, Th17 pathways	Reduce inflammatory amplification	(5, 30, 31)
Microbiome remodeling	Precision decolonization, probiotics, natural products	Key pathogenic and symbiotic bacteria restoration	Break the vicious cycle	(62, 76, 79)
Risk stratification management	Clinical + blood + microbiome models	High-risk identification	Shift from reactive treatment to precision prevention	(11, 14, 67)

SOD, superoxide dismutase; ROS, reactive oxygen species; DAMPs, damage-associated molecular patterns; NETs, neutrophil extracellular traps.

## 5 Prospects and discussion

The understanding of radiation-induced oral mucositis (RIOM) is undergoing a revolution, shifting from a passive epithelial breakdown to a “local ecological civil war” triggered by radiation, immune-dominated, and driven by microbial dysbiosis (54, 62). The “radioimmunomucosology” framework reveals that RIOM is a radiation-induced targeted perturbation of the oral immune ecological niche, and its severity serves as a clinical indicator of local immunopathological activity (8). At the mechanistic level, three interlocking driving loops are activated: In the initiation phase, radiation-induced immunogenic cell death releases DAMPs, activating pathways such as cGAS-STING (6, 39). When efferocytosis fails, secondary necrosis leads to sustained DAMP release, dragging acute inflammation into chronic conditions (38, 43). In the amplification phase, immune cell dysfunction becomes central—excessive NETosis, dysregulated M1/M2 polarization timing, and Th17/Treg imbalance, endowing tissues with “immune memory” (31, 52). In the exacerbation phase, microbes and immunity form a vicious cycle: loss of commensal bacteria lifts inflammatory constraints, Candida drives Th17 differentiation, and key pathogens such as Fusobacterium nucleatum directly amplify inflammation (52, 62, 64). Liao et al. demonstrated that precise eradication of Staphylococcus aureus can halve the risk of severe RIOM, confirming that microbes are active drivers (76).

Zou et al. established a risk prediction model (11), Anderson et al. confirmed that upstream clearance of DAMP precursors can delay the occurrence of RIOM (67), and Liao et al. provided a precise decolonization protocol (76). Together, these three studies outline a multidimensional individualized strategy of “blocking DAMPs upstream, regulating immune imbalance midstream, and clearing key pathogens downstream,” making RIOM a predictable and intervenable immune ecological event (14, 67, 76). A more profound implication is that RIOM can serve as a research model for the tripartite interaction of radiation-immunity-microbiome, with its local disturbances acting as a window into systemic immune status (11, 13, 15). The establishment of “radiation immune mucosology” and the integration of oral ecological management into comprehensive cancer treatment are essential for “optimizing antitumor efficacy while ensuring quality of life” (62, 67). Current evidence supports the adoption of multimodal comprehensive management strategies (102–104), but future research needs to further clarify the optimal intervention timing and combination regimens (61, 105, 106). Nutritional support remains indispensable throughout the treatment process (94, 107).
